# Distribution characteristics and associated factors of dyslipidemia across clinical stages of breast cancer: a retrospective observational study

**DOI:** 10.3389/fgwh.2026.1845647

**Published:** 2026-07-02

**Authors:** Zechang Xin, Hui Qu, Pisong Li, Zhongbin Han, Ningxin Qu, Weiting Yu, Xiaoyu Zhu, Hongshen Chen

**Affiliations:** 1 Department of Breast and Thyroid Surgery, Affiliated Zhongshan Hospital of Dalian University, Dalian, China; 2 Central Laboratory, Affiliated Zhongshan Hospital of Dalian University, Dalian, China

**Keywords:** breast cancer, clinical stage, dyslipidemia, metabolic comorbidity, molecular subtype

## Abstract

**Background:**

Dyslipidemia is a prevalent metabolic disorder in breast cancer patients and an important comorbidity that impacts cardiovascular outcomes, treatment tolerance, and long-term prognosis. It may also be linked to tumor progression, yet the distribution characteristics of lipid abnormalities, especially their heterogeneity across different clinical stages and molecular subtypes of breast cancer, remain insufficiently characterized in existing research.

**Methods:**

This retrospective observational study was conducted at the Affiliated Zhongshan Hospital of Dalian University, enrolling 5,246 patients with pathologically confirmed primary breast cancer diagnosed from 2014 to 2024 with complete clinical staging and fasting lipid profile data. Dyslipidemia was defined as at least one abnormal lipid parameter (LDL-C ≥ 3.4 mmol/L, HDL-C < 1.0 mmol/L, TC ≥ 5.2 mmol/L, TG ≥ 1.7 mmol/L). Stage-stratified multivariable logistic regression analyses were used to identify independent associated factors, with subtype-based subgroup analyses and sensitivity analyses excluding CHD patients performed to verify result robustness.

**Results:**

Dyslipidemia affected 54.8%–71.9% of patients across all clinical stages, with TC and TG abnormalities being the most common, followed by reduced HDL-C and elevated LDL-C. Cumulative proportions of individual lipid abnormalities exceeded 100% per stage, indicating frequent coexistence of multiple abnormalities. Obvious subtype heterogeneity was observed: Luminal B (HER–) had low early-stage HDL-C reduction, while triple-negative patients had a markedly high LDL-C elevation rate (up to 50%) in stage IV. Tumor burden-related factors and selected molecular pathology indicators were independently associated with dyslipidemia in a stage-specific manner, and CHD exclusion did not alter the results.

**Conclusions:**

Dyslipidemia is a pervasive and heterogeneous metabolic comorbidity in breast cancer, with distinct stage- and subtype-specific patterns closely linked to tumor burden and molecular biological characteristics. Routine lipid assessment and subtype-tailored metabolic management should be integrated as an essential component into the comprehensive clinical care of breast cancer patients.

## Introduction

Breast cancer is the most commonly diagnosed malignancy among women worldwide (1). Advances in screening strategies and multimodal treatment approaches have substantially improved survival outcomes, leading to a growing population of long-term breast cancer survivors (2). Consequently, increasing attention has shifted toward comorbid conditions that may influence treatment tolerance, long-term prognosis, and overall quality of life beyond tumor control alone (3).

Dyslipidemia, characterized by abnormal serum lipid levels, is a well-established risk factor for cardiovascular disease and has also been increasingly implicated in cancer biology (4–6). Lipids play essential roles in membrane biosynthesis, energy metabolism, and intracellular signaling, and dysregulated lipid metabolism may facilitate tumor cell proliferation, invasion, and metastasis (7, 8). In breast cancer, lipid metabolism is closely intertwined with estrogen signaling, inflammatory pathways, and tumor microenvironment remodeling (9). Accumulating evidence suggests that lipid abnormalities are common among breast cancer patients; however, most prior studies have focused on isolated lipid parameters or overall prevalence, without systematically examining how different lipid abnormalities are distributed across clinical stages or molecular subtypes (10).

Clinical stage reflects tumor burden and disease aggressiveness, whereas molecular subtype captures intrinsic tumor biology and therapeutic responsiveness (11). Both factors may profoundly influence host metabolic status. Furthermore, dyslipidemia frequently manifests as multiple concurrent lipid abnormalities rather than a single abnormal parameter, suggesting a complex and multidimensional metabolic phenotype (12). Despite its potential clinical relevance, the stage- and subtype-specific distribution characteristics of dyslipidemia in breast cancer, as well as the associated clinical and pathological determinants, remain poorly defined. Therefore, the present study aimed to comprehensively characterize the distribution patterns of dyslipidemia across clinical stages and breast cancer subtypes, identify stage-specific associated factors, and evaluate the robustness of these findings through sensitivity analyses.

## Materials and methods

### Study design and population

**Figure 1:**
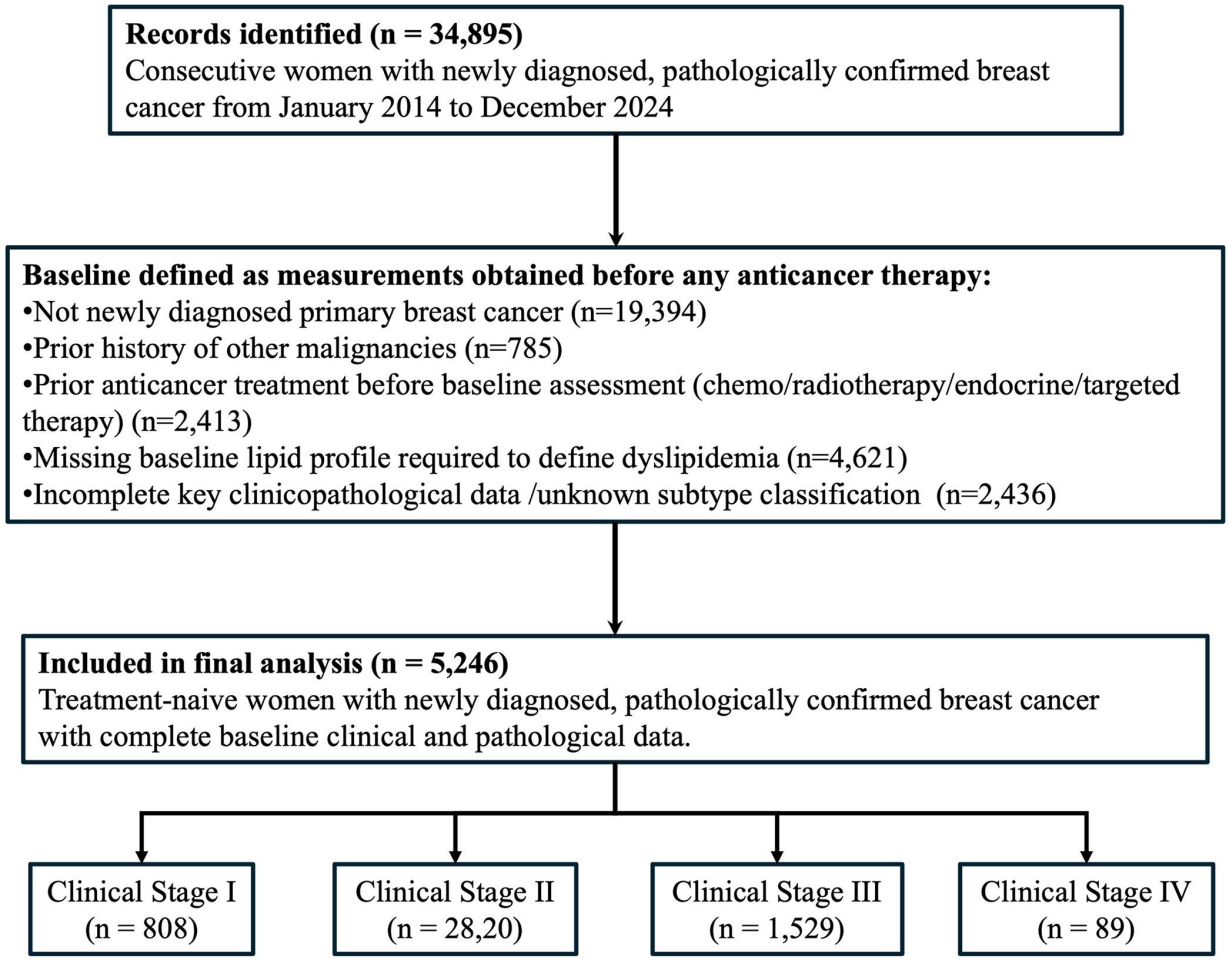
Study flowchart of breast cancer patients included in the dyslipidemia analysis.

This retrospective observational study was conducted at The Affiliated Zhongshan Hospital of Dalian University, a tertiary medical center. Female patients with pathologically confirmed primary breast cancer diagnosed at this hospital between January 2014 and December 2024 were eligible for inclusion. Clinical stage was determined at diagnosis according to the TNM classification system. All enrolled patients, regardless of receiving upfront surgery or neoadjuvant therapy, were staged in accordance with the unified AJCC TNM staging criteria. For patients who experienced pathological upstaging after surgical resection (e.g., clinical N0 converted to pathological N1), the final postoperative pathological stage was adopted as the definitive clinical stage for all stratified analyses. A detailed staging decision flow diagram is provided below to illustrate the process of integrating clinical and pathological findings for stage assignment. Patients were excluded if fasting lipid profile data were unavailable or incomplete, or if essential clinical or pathological information required for analysis was missing. After applying inclusion and exclusion criteria, a total of 5,246 patients were included in the final analysis (Figure 1). The study protocol was reviewed and approved by the Ethics Committee of The Affiliated Zhongshan Hospital of Dalian University(Approval No.: PJ-KY2026-016-1). Owing to the retrospective design of the study and the use of anonymized data, the requirement for informed consent was waived.

### Data collection

Demographic information, including age at diagnosis, was obtained from medical records. Comorbid conditions such as hypertension, diabetes mellitus, and coronary heart disease were identified based on documented medical history at admission. Tumor-related variables included clinical stage, lymph node metastasis, intravascular tumor thrombus, and breast cancer subtype, all of which were determined according to pathological and clinical records.

Molecular pathology indicators, including E-cadherin, P120 catenin, TOPII, and CK5/6 expression, were obtained from pathology reports and assessed using standard immunohistochemical criteria. Biochemical indicators, including serum creatinine, uric acid, and body mass index (BMI), were collected at baseline prior to the initiation of cancer-specific treatment.

Serum lipid profiles, including low-density lipoprotein cholesterol (LDL-C), high-density lipoprotein cholesterol (HDL-C), total cholesterol (TC), and triglycerides (TG), were measured using fasting blood samples collected at diagnosis. Dyslipidemia was defined as at least one abnormal lipid parameter (LDL-C ≥ 3.4 mmol/L, HDL-C < 1.0 mmol/L, TC ≥ 5.2 mmol/L, TG ≥ 1.7 mmol/L). All cut-off values for lipid indicators were determined in line with the Chinese Guidelines for the Management of Dyslipidemia in Adults (13).

### Statistical analysis

Continuous variables are presented as mean ± standard deviation, and categorical variables as counts and percentages. Differences in baseline characteristics and lipid abnormalities across clinical stages were assessed using analysis of variance for continuous variables and chi-square tests for categorical variables. Stage-stratified multivariable logistic regression analyses were performed to identify factors independently associated with dyslipidemia.

Variables included in the multivariable models were selected based on clinical relevance and univariable screening results. Subgroup analyses were conducted according to breast cancer subtype. Sensitivity analyses excluding patients with a history of coronary heart disease were performed to assess robustness. Odds ratios (ORs) with corresponding 95% confidence intervals (CIs) were calculated. A two-sided *P* value < 0.05 was considered statistically significant. All analyses were conducted using R software.

## Results

### Baseline characteristics of the study population

The baseline demographic, clinical, and pathological characteristics of the study population stratified by clinical stage are summarized in Table 1. The mean age of patients was comparable across stages, with no clear age gradient observed from stage I to stage IV. The prevalence of common metabolic comorbidities, including hypertension and diabetes mellitus, was generally similar across stages, indicating a relatively balanced baseline metabolic profile.

**Table 1:** The baseline characteristics of the participants.

Characteristic	Clinical stage I (*n* = 808)	Clinical stage II (*n* = 2,820)	Clinical stage III (*n* = 1,529)	Clinical stage IV (*n* = 89)	Total (*n* = 5,246)	*P* value
Age	55.41 ± 9.31	55.66 ± 9.38	54.89 ± 9.14	56.4 ± 9.04	55.41 ± 9.3	0.048
Hypertension	48 (5.9%)	146 (5.2%)	106 (6.9%)	6 (6.7%)	306 (5.8%)	0.126
Diabetes mellitus	26 (3.2%)	62 (2.2%)	34 (2.2%)	2 (2.2%)	124 (2.4%)	0.388
CHD	42 (5.2%)	120 (4.3%)	78 (5.1%)	6 (6.7%)	246 (4.7%)	0.38
Electrocardiographic abnormalities						
Any arrhythmia	11 (1.4%)	28 (1.0%)	11 (0.7%)	3 (3.4%)	53 (1.0%)	0.064
Sinus bradycardia	22 (2.7%)	47 (1.7%)	15 (1.0%)	1 (1.1%)	85 (1.6%)	0.017
Sinus tachycardia	2 (0.2%)	7 (0.2%)	4 (0.3%)	1 (1.1%)	14 (0.3%)	0.475
Sinus arrhythmia	5 (0.6%)	6 (0.2%)	0 (0.0%)	2 (2.2%)	13 (0.2%)	<0.001
Atrial fibrillation	3 (0.4%)	4 (0.1%)	3 (0.2%)	0 (0.0%)	10 (0.2%)	0.591
Atrioventricular block	2 (0.2%)	13 (0.5%)	4 (0.3%)	0 (0.0%)	19 (0.4%)	0.613
Breast cancer type						<0.001
LuminalA	390 (48.3%)	1,393 (49.4%)	586 (38.3%)	36 (40.4%)	2,405 (45.8%)	
Luminal B (HER2⁻)	199 (24.6%)	539 (19.1%)	388 (25.4%)	22 (24.7%)	1,148 (21.9%)	
Luminal B (HER2⁺)	53 (6.6%)	249 (8.8%)	156 (10.2%)	9 (10.1%)	467 (8.9%)	
HER2-enriched	78 (9.7%)	325 (11.5%)	203 (13.3%)	14 (15.7%)	620 (11.8%)	
Triple Negative	88 (10.9%)	314 (11.1%)	196 (12.8%)	8 (9.0%)	606 (11.6%)	
Tumor Pathology & Staging Indicators						
Lymph node metastasis	0 (0%)	1,977 (70.01%)	1,491 (97.5%)	85 (95.5%)	3,553 (67.7%)	<0.001
Intravascular tumor thrombus	386 (47.8%)	1,815 (64.4%)	811 (53.0%)	47 (52.8%)	3,059 (58.3%)	<0.001
T						<0.001
1	808 (100%)	862 (30.6%)	176 (11.5%)	5 (5.6%)	1,851 (35.3%)	
2	0 (0.0%)	1,898 (67.3%)	343 (22.4%)	18 (20.2%)	2,259 (43.1%)	
3	0 (0.0%)	60 (2.1%)	525 (34.3%)	15 (16.9%)	600 (11.4%)	
4	0 (0.0%)	0 (0.0%)	485 (31.7%)	51 (57.3%)	536 (10.2%)	
N						<0.001
0	808 (100%)	843 (29.9%)	38 (2.5%)	4 (4.5%)	1,693 (32.3%)	
1	0 (0.0%)	1,977 (70.1%)	144 (9.4%)	13 (14.6%)	2,134 (40.7%)	
2	0 (0.0%)	0 (0.0%)	781 (51.1%)	19 (21.3%)	800 (15.2%)	
3	0 (0.0%)	0 (0.0%)	566 (37.0%)	53 (59.6%)	619 (11.8%)	
M						<0.001
1	0 (0.0%)	0 (0.0%)	0 (0.0%)	89 (100.0%)	89 (1.7%)	
Molecular pathology indicators						
E-cadherin	147 (18.2%)	467 (16.6%)	254 (16.6%)	22 (24.7%)	890 (17.0%)	0.1
TOPOII	406 (50.2%)	1,348 (47.8%)	705 (46.1%)	50 (56.2%)	2,509 (47.8%)	0.067
P120 catenin	345 (42.7%)	1,134 (40.2%)	517 (33.8%)	46 (51.7%)	2,042 (38.9%)	<0.001
CK5/6	183 (22.6%)	755 (26.8%)	388 (25.4%)	27 (30.3%)	1,353 (25.8%)	0.06
Biochemical & lipid indicators						
Dyslipidemia	443 (54.8%)	202 (71.6%)	1,040 (68.0%)	65 (73.0%)	3,568 (68.0%)	<0.001
LDL (mmol/L)	2.75 ± 1.36	3.04 ± 1.56	2.89 ± 1.47	2.68 ± 1.26	2.95 ± 1.51	<0.001
HDL (mmol/L)	1.18 ± 0.35	1.13 ± 0.38	1.16 ± 0.37	1.11 ± 0.45	1.14 ± 0.38	0.001
Total cholesterol (mmol/L)	3.78 ± 1.71	3.94 ± 1.77	4.04 ± 1.77	4.01 ± 1.86	3.95 ± 1.77	0.006
Total triglycerides (mmol/L)	1.65 ± 0.98	1.8 ± 1.14	1.72 ± 1.07	1.79 ± 1.04	1.75 ± 1.1	0.003
Hormone & renal function indicators						
Estradiol (pg/mL)	209.92 ± 117.96	196.91 ± 116.29	202.83 ± 113.03	194.57 ± 115.41	200.59 ± 115.66	0.036
Follicle-stimulating hormone (mIU/mL)	95.38 ± 54.52	102.55 ± 55.13	102.00 ± 54.79	100.65 ± 55.75	101.25 ± 54.99	0.011
Serum creatinine(*μ*mol/L)	89.64 ± 54.52	92.57 ± 51.46	86.84 ± 50.01	88.25 ± 50.79	90.37 ± 51.57	0.005
Uric acid(μmol/L)	266.03 ± 120.00	269.41 ± 124.62	270.54 ± 121.19	292.97 ± 113.33	269.62 ± 122.75	0.261
BMI (kg/m²)	24.49 ± 2.86	24.37 ± 2.65	24.50 ± 2.71	23.87 ± 2.48	24.42 ± 2.7	0.096
Tumor markers						
CEA (ng/mL)	9.68 ± 20.11	8.89 ± 19.68	8.65 ± 10.72	8.12 ± 6.44	8.93 ± 17.46	0.559
CA125(U/mL)	64.56 ± 54.91	69.48 ± 71.18	68.76 ± 63.35	64.8 ± 56.07	68.43 ± 66.43	0.29
CA153 (U/mL)	29.69 ± 44.79	28.54 ± 43.37	28.44 ± 44.41	31.33 ± 47.97	28.74 ± 43.97	0.846

In contrast, tumor-related characteristics demonstrated pronounced stage-dependent distributions. The proportions of patients with lymph node metastasis and intravascular tumor thrombus increased markedly with advancing clinical stage, reflecting progressive tumor burden and disease severity. The distribution of breast cancer subtypes also differed significantly across stages, highlighting heterogeneity in tumor biology across the disease spectrum.

### Serum lipid profiles across clinical stages

Serum lipid profiles according to clinical stage are presented in Table 2. Across all stages, abnormalities in total cholesterol and triglycerides were the most frequently observed lipid disorders. The proportions of patients with borderline-high or high TC and TG levels remained consistently elevated from early-stage to advanced-stage disease, suggesting widespread dysregulation of lipid synthesis and storage throughout the disease course.

**Table 2:** The serum lipid control among breast cancer patients.

Characteristic	Clinical stage I	Clinical stage II	Clinical stage III	Clinical stage IV	Total	*P* value
LDL (mmol/L)						<0.001
Ideal level, LDL < 2.6	425 (52.9%)	1,288 (45.8%)	751 (49.2%)	46 (52.9%)	2,510 (48.0%)	
Appropriate level, 2.6 ≤ LDL < 3.4	239 (29.8%)	783 (27.8%)	449 (29.4%)	24 (27.6%)	1,506 (28.7%)	
Borderline high, 3.4 ≤ LDL < 4.1	26 (3.2%)	136 (4.8%)	66 (4.3%)	5 (5.7%)	233 (4.5%)	
High LDL ≥ 4.1	113 (14.1%)	607 (21.6%)	259 (17%)	12 (13.8%)	991 (19%)	
HDL (mmol/L)						0.001
Low HDL < 1	751 (21.0%)	128 (17.4%)	160 (18.7%)	20 (22.5%)	1,059 (20.2%)	
TC (mmol/L)						0.001
Appropriate level, TC < 5.2	633 (78.3%)	2,051 (72.7%)	1,073 (70.2%)	62 (69.7%)	3,819 (72.8%)	
Borderline high, 5.2 ≤ TC < 6.2	85 (10.5%)	386 (13.7%)	248 (16.2%)	12 (13.5%)	731 (13.9%)	
High level, TC ≥ 6.2	90 (11.1%)	383 (13.6%)	208 (13.6%)	15 (16.9%)	696 (13.3%)	
TG (mmol/L)						0.003
Appropriate level, TG < 1.7	633 (78.3%)	2,066 (75.6%)	1,134 (76.3%)	64 (71.9%)	3,897 (74.3%)	
Borderline high, 1.7 ≤ TG < 2.3	39 (4.8%)	206 (7.3%)	138 (9%)	7 (7.9%)	390 (7.4%)	
High level, TG ≥ 2.3	136 (16.8%)	548(19.4%)	257(16.8%)	18(20.2%)	959(18.7%)	

In contrast, tumor-related characteristics demonstrated pronounced stage-dependent distributions. The proportions of patients with lymph node metastasis and intravascular tumor thrombus increased markedly with advancing clinical stage, reflecting progressive tumor burden and disease severity. The distribution of breast cancer subtypes also dif-fered significantly across stages, highlighting heterogeneity in tumor biology across the disease spectrum.

### Distribution characteristics of dyslipidemia across clinical stages

**Figure 2:**
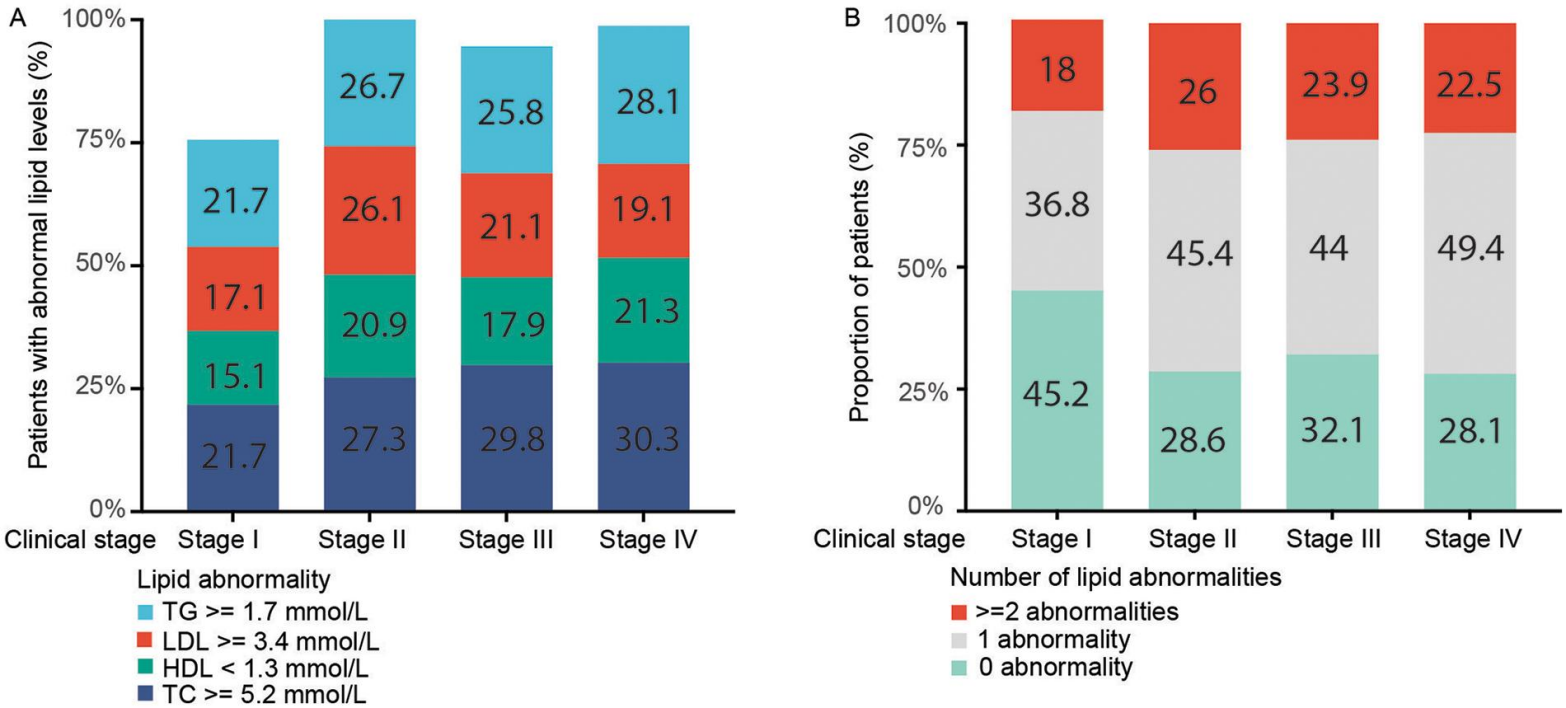
Distribution characteristics of dyslipidemia across clinical stages **(A)** distribution of individual lipid abnormalities by clinical stage in breast cancer patients. **(B)** Distribution of the number of lipid abnormalities by clinical stage in breast cancer patients. All percentage values shown in Panel A and Panel B are row percentages calculated within each corresponding clinical stage. Owing to the stacked segmented layout, error bars were not plotted; sample sizes differ across clinical stages.

The distribution characteristics of dyslipidemia across clinical stages are illustrated in Figures 2. As shown in Figure 2A, TC and TG abnormalities constituted the dominant lipid disorders across all stages, followed by reduced HDL-C and elevated LDL-C. Many patients exhibited concurrent multiple lipid abnormalities, representing a metabolically high-risk multimorbid profile with important clinical implications for breast cancer management.

Figure 2B further demonstrates the distribution of patients according to the number of lipid abnormalities across clinical stages. Dyslipidemia, defined as the presence of at least one abnormal lipid parameter, was highly prevalent even in early-stage disease. Approximately one-quarter of patients presented with two or more concurrent lipid abnormalities across all stages, while the proportion of patients without any lipid abnormality declined modestly with advancing stage. Together, these findings highlight the multidimensional nature of dyslipidemia in breast cancer patients.

### Subtype-specific lipid abnormality patterns

Subtype-specific analyses revealed distinct lipid abnormality patterns across clinical stages (Table 3). Luminal B (HER–) patients exhibited a notably low prevalence of reduced HDL-C in early stages, whereas Luminal B (HER+) patients demonstrated increasing TC abnormality prevalence with advancing stage. HER2-enriched tumors showed moderate lipid abnormality rates without a clear stage-dependent trend.

**Table 3:** Lipid abnormality prevalence by breast cancer subtype and clinical stage.

Breast cancer type	Clinical stage	Elevated TC (%)	Elevated TG (%)	Decreased HDL-C (%)	Elevated LDL-C (%)
Luminal A	I	22.3	25.6	18.5	21.8
Luminal A	II	23	28.8	26.3	34
Luminal A	III	24.2	25.8	24.1	31.9
Luminal A	IV	25	36.1	22.2	22.2
Luminal B (HER-)	I	23.1	20.6	9	5
Luminal B (HER-)	II	35.3	24.1	9.3	8.3
Luminal B (HER-)	III	38.4	21.9	7	4.9
Luminal B (HER-)	IV	36.4	31.8	22.7	4.5
Luminal B (HER+)	I	15.1	13.2	18.9	18.9
Luminal B (HER+)	II	28.1	24.1	16.5	22.5
Luminal B (HER+)	III	34	37.2	24.4	22.4
Luminal B (HER+)	IV	44.4	33.3	11.1	11.1
HER2-enriched	I	11.5	15.4	14.1	17.9
HER2-enriched	II	30.2	23.1	23.1	26.5
HER2-enriched	III	26.6	25.6	21.2	22.2
HER2-enriched	IV	28.6	7.1	21.4	21.4
Triple Negative	I	28.4	17	12.5	21.6
Triple Negative	II	30	28	18.2	24.5
Triple Negative	III	29.6	25	12.8	18.4
Triple Negative	IV	25	12.5	25	50

Data are presented as percentage of patients with abnormal lipid pa-rameters (TC ≥ 5.2 mmol/L, TG ≥ 1.7 mmol/L, HDL < 1.3 mmol/L, LDL ≥ 3.4 mmol/L) in each subtype-stage subgroup. TC, total cholesterol; TG, triglycerides; HDL, high-density lipoprotein cholesterol; LDL, low-density lipoprotein cholesterol.

In contrast, Triple Negative breast cancer exhibited a strikingly high prevalence of elevated LDL-C in stage IV disease, reaching 50%. This pattern suggests a potential association between aggressive tumor biology and dysregulated cholesterol metabolism in this subtype.

### Sensitivity analysis excluding CHD patients

To evaluate the robustness of dyslipidemia prevalence estimates, a sensitivity analysis excluding patients with a history of coronary heart disease was performed. As shown in Table 4, dyslipidemia prevalence across clinical stages changed minimally after exclusion, with a maximum difference of 0.8%. These results indicate that the observed high prevalence of dyslipidemia was not driven by pre-existing cardiovascular disease.

**Table 4:** Sensitivity analysis of dyslipidemia prevalence after excluding patients with coronary heart disease (CHD) history.

Clinical stage	Original prevalence (%)	Prevalence (excl. CHD) (%)
I	54.8	55.2
II	71.4	71.5
III	67.9	68
IV	71.9	71.1

Data are presented as percentage of patients with dyslipidemia (≥1 ab-normal lipid parameter) in the original cohort and after excluding patients with a history of CHD.

### Factors associated with dyslipidemia

To identify the independent clinical and pathological determinants of dyslipidemia across disease progression, we performed stage-stratified multivariable logistic regression analyses (Table 5). These analyses revealed that age, tumor burden-related characteristics, and select molecular pathology indicators were independently associated with dyslipidemia, with associations varying by clinical stage.

**Table 5:** Associated factors of dyslipidemia among breast cancer patients.

Characteristic	Clinical stage I			Clinical stage II			Clinical stage III			Clinical stage IV		
	*P* value	OR	95% C.I.	*P* value	OR	95% C.I.	*P* value	OR	95% C.I.	*P* value	OR	95% C.I.
Age	0.107	1.015	0.997–1.035	<0.001	1.025	1.014–1.035	<0.001	1.025	1.011–1.039	0.848	1.009	0.925–1.1
Hypertension	0.867	0.913	0.313–2.66	0.382	0.771	0.43–1.382	0.222	1.609	0.75–3.449	1	–	–
Diabetes mellitus	0.831	0.883	0.283–2.757	0.223	0.631	0.3–1.324	0.134	0.511	0.213–1.229	1	–	–
Breast cancer type	<0.001	0.756	0.651–0.878	0.001	0.852	0.7778–0.932	0.01	0.833	0.749–0.927	0.174	0.444	0.138–1.43
CHD	0.522	0.695	0.228–2.117	0.337	1.392	0.708–2.738	0.44	0.719	0.311–1.661	0.999	–	–
Electrocardiographic abnormalities												
Any arrhythmia	0.941	0.943	0.199–4.463	0.16	0.5	0.10–1.315	–	–	–	0.999	–	–
Tumor pathology & staging indicators												
Lymph node metastasis	<0.001	2.046	1.736–2.412	<0.001	2.402	1.976–2.92	0.002	3.423	1.559–7.515	0.856	1.449	0.026–79.558
Intravascular tumor thrombus	<0.001	3.231	2.263–4.614	<0.001	3.689	2.992–4.549	<0.001	2.35	1.809–3.052	0.226	2.892	0.519–16.122
Molecular pathology indicators												
E-cadherin	0.002	0.42	0.245–0.722	<0.001	0.427	0.309–0.59	0.006	0.584	0.397–0.857	0.856	1.253	0.11–14.307
TOPOII	0.453	1.152	0.796–1.667	0.617	1.059	0.846–1.324	0.291	1.157	0.882–1.519	0.763	1.352	0.191–9.565
P120 catenin	0.589	1.108	0.764–1.606	0.019	1.301	1.044–1.621	0.078	1.285	0.973–1.697	0.174	0.288	0.048–1.731
CK5/6	0.758	1.078	0.667–1.743	0.238	0.841	0.631–1.121	0.408	1.167	0.81–1.682	0.631	2.272	0.08–64.654
Hormone & renal function indicators												
Estradiol (pg/mL)	0.668	1	0.999–1.002	0.386	1	0.999–1.000	0.411	1	0.999–1.001	0.128	1.005	0.998–1.012
Follicle-stimulating hormone (mIU/mL)	0.791	1	0.999–1.003	0.263	1.001	0.999–1.003	0.169	1.002	1–1.002	0.218	1.008	0.995–1.020
Serum creatinine	0.152	1.002	0.999–1.005	0.259	1.001	0.999–1.003	0.995	1	0.997–1.004	0.215	0.991	0.976–1.005
Uric acid	0.441	1.001	0.999–1.002	0.024	1.001	1.000–1.002	0.277	1.001	1–1.002	0.157	0.995	0.989–1.002
BMI (kg/m²)	0.445	0.975	0.915–1.04	0.383	1.018	0.978–1.059	0.319	1.026	0.976–1.078	0.039	1.508	1.021–2.228
Tumor markers												
CEA (Carcinoembryonic antigen)	0.451	1.006	0.993–1.019	0.897	1.001	0.989–1.012	0.841	1.001	0.990–1.013	0.672	1.025	0.913–1.151
CA125	0.451	0.999	0.996–1.002	0.033	0.998	0.997–1.000	0.96	1	0.998–1.002	0.024	0.986	0.974–0.998
CA153	0.395	0.998	0.994–1.002	0.812	1	0.998–1.003	0.72	1.001	0.998–1.004	0.657	1.005	0.985–1.025

“–”: Statistical results were unstable (e.g., insufficient sample size in the subgroup) and thus not reported. CHD, coronary heart disease; BMI, body mass index; CEA, carcinoembryonic antigen.

Age was inversely associated with dyslipidemia across stages I–III. Tumor burden–related factors, including lymph node metastasis and intravascular tumor thrombus, were consistently associated with increased odds of dyslipidemia across multiple stages. Molecular pathology indicators demonstrated stage-specific associations. For example, E-cadherin expression was inversely associated with dyslipidemia in early-stage disease, whereas these associations were attenuated in advanced stages. Associations observed in stage IV disease should be interpreted cautiously due to the relatively small sample size.

## Discussion

In this large retrospective study, we comprehensively characterized the distribution patterns of dyslipidemia across clinical stages and molecular subtypes of breast cancer, and identified stage-specific associated factors using stratified multivariable regression analyses. Dyslipidemia was highly prevalent across all stages of breast cancer, particularly abnormalities in total cholesterol (TC) and triglycerides (TG). These findings highlight that dysregulated lipid metabolism is common in breast cancer, consistent with growing recognition of lipids as important metabolic components in tumor biology rather than merely late-stage complications.

### Comparison with prior studies and novel insights

The high prevalence of dyslipidemia in our cohort aligns with existing literature emphasizing the importance of lipid alterations in breast cancer patients. Previous work has shown that lipid metabolic abnormalities are linked to tumor progression, treatment response, and prognosis, with elevated LDL-C and TG levels associated with more aggressive disease features and poor outcomes in breast cancer survivors (10). Dyslipidemia may influence breast cancer progression and long-term prognosis, including chemotherapy-induced changes in lipid profiles that persist post-treatment and contribute to increased cardiovascular risks in survivors.

Most prior studies, however, have focused on overall prevalence or post-treatment changes in dyslipidemia rather than a systematic comparison across clinical stages and molecular subtypes. Our stage-stratified analysis reveals that dyslipidemia is pervasive across all stages, not confined to advanced disease, suggesting intrinsic metabolic reprogramming in breast cancer biology. Furthermore, our subtype-specific analysis revealed heterogeneity in lipid profiles across molecular subtypes. For example, Triple Negative breast cancer (TNBC) patients exhibited a notably high prevalence of elevated LDL-C in stage IV disease, whereas Luminal subtypes displayed differing patterns of HDL-C and TC abnormalities (14). These subtype-specific features echo emerging evidence that metabolic profiles vary with tumor biology, but fine-grained clinical-stage comparisons have been lacking in the literature. We defined dyslipidemia based on the Chinese Guidelines for the Management of Dyslipidemia in Adults, and interpreted distinct clinical implications of each lipid indicator accordingly (13).

### Mechanistic implications of lipid metabolism in breast cancer

Lipid metabolism plays multifaceted roles in breast cancer progression. Clinical and experimental evidence supports that lipids are not passive biomarkers but actively participate in cancer cell proliferation, signaling, and microenvironment modulation (15). Elevated circulating lipids may provide essential structural components for rapidly proliferating tumor cells and serve as energy reservoirs (16). Alterations in cholesterol and fatty acid metabolism have been implicated in membrane biosynthesis, signal transduction cascades, and modulation of tumor-promoting immune environments (16, 17).

Inflammatory processes linked to aggressive tumors may contribute to systemic lipid disturbances. Tumor cells and associated stromal elements can secrete pro-inflammatory cytokines, which influence hepatic lipid metabolism and favor elevated TC and TG levels. Additionally, obesity-associated lipid handling pathways—such as enhanced fatty acid transport and storage—have been linked to aggressive phenotypes in breast cancer, including TNBC, where dysregulated lipid uptake and utilization are proposed contributors to metastatic potential (18–20).

The markedly elevated LDL-C rate reaching 50% among patients with stage IV TNBC reflects multifactorial disparities between breast cancer molecular subtypes. Endogenous estrogen receptor signaling exerts profound regulatory effects on systemic lipid homeostasis: luminal subtype tumors retain functional ER pathways that constrain hepatic cholesterol synthesis and facilitate LDL clearance, whereas TNBC lacks intact ER expression and loses this intrinsic lipid-protective regulatory network (21). Beyond biological variance, disparities in treatment regimens introduce potential confounding effects; first-line chemotherapy for TNBC frequently incorporates corticosteroid agents, a class widely documented to raise circulating LDL-C and disrupt lipid profiles during anti-tumor therapy (22).

The observed stage-specific association between molecular markers (e.g., E-cadherin) and dyslipidemia may reflect changes in tumor cell biology across progression. E-cadherin loss during epithelial-mesenchymal transition (EMT) is known to coincide with increased invasiveness and metastatic behavior, and dysregulated lipid metabolism may further support this transition by altering membrane dynamics and cellular signaling networks. Although the precise molecular pathways linking changes in adhesion proteins to host lipid profiles require further investigation, the dynamic associations we observed suggest that lipid metabolism may be influenced by, and in turn influence, key tumor phenotypes.

### Clinical implications and future directions

Our findings have clear clinical implications. Current breast cancer management guidelines, including lipid monitoring protocols, often focus on patients receiving endocrine therapy due to the known impact of aromatase inhibitors on lipid profiles. However, our data indicate that dyslipidemia is common even in early-stage patients irrespective of endocrine therapy exposure. This suggests that routine lipid screening should be considered at the time of breast cancer diagnosis, not solely in patients flagged for treatment-related risk.

Subtype-specific patterns further imply that lipid management may benefit from a tailored approach. For instance, elevated LDL-C in advanced-stage TNBC suggests a potential role for therapeutic interventions targeting cholesterol metabolism. While randomized controlled trials specifically assessing statin or other lipid-lowering interventions in breast cancer cohorts remain limited, preclinical data and observational findings support potential anticancer benefits of targeting lipid pathways, in addition to cardiovascular risk reduction (10).

The frequent coexistence of multiple lipid abnormalities (∼25% of patients across stages) also warrants attention, as combined lipid disturbances are associated with higher cardiovascular event risk in the general population. Although direct evidence linking multiple concurrent lipid abnormalities to breast cancer-specific outcomes remains limited, optimizing lipid profiles may confer dual benefits for both cardiovascular health and cancer prognosis in survivorship care programs.

### Limitations

Several limitations should be acknowledged. This retrospective study cannot establish causal relationships, and lipid profiles were only measured at a single time point, so dynamic changes during treatment could not be evaluated. We lacked data on lipid-lowering medications, menopausal status, dietary habits and physical activity—all important factors affecting serum lipids. Although estradiol and follicle-stimulating hormone were detected, these markers were not used for menopausal subgroup analysis, and we were unable to conduct a sensitivity analysis excluding patients taking lipid-modifying drugs. Moreover, waist circumference and waist-hip ratio, more sensitive metabolic risk indicators, were not collected. The small sample size of stage IV patients (*n* = 89) may lead to wide confidence intervals, and the observed BMI correlation in this subgroup should be interpreted with caution. Notably, all BMI data were objectively measured at baseline rather than self-reported. Despite these limitations, the large sample size and detailed stratification by clinical stage and molecular subtype strengthen the reliability of our results.

## Conclusions

Dyslipidemia is a highly prevalent, stage- and subtype-heterogeneous metabolic comorbidity in breast cancer, closely associated with tumor burden and biological characteristics. Our cross-sectional observations raise the hypothesis that routine lipid assessment could be considered within comprehensive breast cancer management, yet prospective studies with cardiovascular and survival endpoints are required to confirm its clinical value. Future prospective studies are needed to clarify causal mechanisms linking tumor subtype to lipid dysregulation and to determine whether targeted lipid-modifying interventions can improve both metabolic and oncologic outcomes.

## Data Availability

The raw data supporting the conclusions of this article will be made available by the authors, without undue reservation.
